# All-or-none neural mechanisms underlying face categorization: evidence from the N170

**DOI:** 10.1093/cercor/bhac101

**Published:** 2022-03-15

**Authors:** Haiyang Jin, William G Hayward, Paul M Corballis

**Affiliations:** School of Psychology, University of Auckland, 23 Symonds Street, Auckland Central, Auckland, 1010, New Zealand; Centre for Brain Research, University of Auckland, 85 Park Road, Grafton, Auckland 1023, New Zealand; Department of Psychology, New York University Abu Dhabi, PO Box 129188, Saadiyat Island, Abu Dhabi, United Arab Emirates; Department of Psychology, University of Hong Kong, Centennial Campus, Pokfulam Road, Hong Kong, China; School of Psychology, University of Auckland, 23 Symonds Street, Auckland Central, Auckland, 1010, New Zealand; Centre for Brain Research, University of Auckland, 85 Park Road, Grafton, Auckland 1023, New Zealand

**Keywords:** equivalence tests, event-related potentials, face categorization, graded responses, subjective perception

## Abstract

Categorization of visual stimuli is an intrinsic aspect of human perception. Whether the cortical mechanisms underlying categorization operate in an all-or-none or graded fashion remains unclear. In this study, we addressed this issue in the context of the face-specific N170. Specifically, we investigated whether N170 amplitudes grade with the amount of face information available in an image, or a full response is generated whenever a face is perceived. We employed linear mixed-effects modeling to inspect the dependency of N170 amplitudes on stimulus properties and duration, and their relationships to participants’ subjective perception. Consistent with previous studies, we found a stronger N170 evoked by faces presented for longer durations. However, further analysis with equivalence tests revealed that this duration effect was eliminated when only faces perceived with high confidence were considered. Therefore, previous evidence supporting the graded hypothesis is more likely to be an artifact of mixing heterogeneous “all” and “none” trial types in signal averaging. These results support the hypothesis that the N170 is generated in an all-or-none manner and, by extension, suggest that categorization of faces may follow a similar pattern.

## Introduction

The ability to categorize environmental stimuli is a fundamental function of human perceptual systems. Perceptual categorization allows us to organize rich and dynamic sensory information from the environment and supports higher-level cognitive processes such as learning and memory (Retter et al. 2020). A critical question is whether the neural mechanisms subserving categorization function operates in a graded or all-or-none fashion. That is, do these system responses grade with the amount of evidence for some particular category, or do they operate in a more binary fashion, generating a full-strength response only when the evidence for that category passes some threshold? Addressing this question will deepen our understanding of the brain basis for categorization and more broadly will advance the construction of valid theoretical models.

One canonical neural signature of perceptual categorization in the visual system is the face-specific enhancement of the N1 component of the event-related potential (ERP)—typically termed the N170 (e.g. Bentin et al. 1996). The N170 is scalp negativity that is maximal at posterior electrode locations in response to the presentation of a face image. As the nomenclature suggests, the peak amplitude of this component typically occurs around 170 ms poststimulus. It is the earliest reliable ERP correlate of face perception. While the face-specific N170 has been the subject of intense research for nearly 3 decades (for reviews, see Rossion 2014; Rossion and Jacques 2011; Schweinberger and Neumann 2016), it remains uncertain whether this response is generated in a graded fashion, varying in amplitude as a function of the amount or quality of face information available in an image, or is a full response generated whenever a face is perceived.

The idea that the N170 is graded is supported by the observation that lower N170 (or M170, the equivalent response observed in magnetoencephalography) amplitudes were recorded when face information was reduced by either reducing the exposure duration (Carbon et al. 2005; Tanskanen et al. 2007) or adding noise to the image (Tarkiainen et al. 2002; Jemel et al. 2003; Horovitz et al. 2004; Philiastides et al. 2006; Philiastides and Sajda 2006; Schneider et al. 2007; Rousselet et al. 2008; Bankó et al. 2011; Navajas et al. 2013; Németh et al. 2014). While these reports appear to be consistent with the view that N170 amplitudes are graded with the amount of evidence for a face, on critical evaluation this conclusion may not be warranted. A number of studies of face detection tasks have reported that, in addition to reducing N170 amplitudes, limiting the amount of information available also resulted in poorer behavioral performance (Rousselet et al. 2008; Nasr 2010; Bankó et al. 2011; Retter et al. 2020) and that N170 amplitude also varied positively with behavioral performance (Philiastides and Sajda 2006; Tanskanen et al. 2007; Rousselet et al. 2008; Nasr 2010; Bankó et al. 2011; Eiserbeck et al. 2021). There are two possible explanations for the relationship between the quality of face information, the amplitude of the face-specific N170, and behavioral performance on face categorization tasks. At the single-trial level, reducing the quality of face information in the image may elicit a weaker “face” response—and thus a lower-amplitude N170 compared to trials with higher-quality images (i.e. the N170 is a graded response reflecting the strength of the categorization response), so the average N170 across trials is commensurately lower in amplitude. Alternatively, the relationship between face information and N170 amplitude could be an artifact of the signal averaging inherent in extracting visual evoked potentials. That is, a full-strength N170 may be generated on every trial on which a face is perceived (i.e. the N170 is an all-or-none response reflecting the perception of a face), but since fewer faces are detected on trials with reduced face information, signal averaging results in an N170 that is lower in amplitude for these trials than it is for trials with more face information. While some studies tried to resolve these possibilities by only computing N170 for trials in which a face was correctly perceived (Fisch et al. 2009; Rodríguez et al. 2012; Navajas et al. 2013), it remains possible that the N170 amplitude might still be biased by correct guessing in these studies. To distinguish more completely between these possibilities, it is critical to take both behavioral performance and signal averaging across trials into account.

To disentangle these alternatives, we employed linear mixed modeling (LMM) with null hypothesis significance testing (NHST) and equivalence tests (ETs) to examine the dependency of the N170 on face and house images with different durations while participants reported subjective confidence on each trial. Several measures were taken to ensure that participants did have a clear perception of faces when they reported high subjective confidence. First, the instructions emphasized to participants to press specific keys (e.g. Key 1 when participants were sure, the stimulus was a face) only when they were sure what the stimulus was, otherwise use other keys (more see Procedure). Second, the percentage of stimuli being intact faces when participants responded with Key 1 in the 33 ms condition was assessed to make sure that participants followed the instruction. Participants who failed to correctly respond on at least 95% of these trials were excluded from further analyses. Third, all stimuli were presented for 33 ms in the first half of the experiment to eliminate the possibility that participants were inclined to report lower confidence for faces presented for 33 ms but high confidence for faces presented for 216 ms even when participants were sure the stimulus was a face in both conditions. Finally, preceding the second half of experiment where stimuli were presented for 33 ms or 216 ms, participants were informed of the 216 ms condition and instructed to keep the same criteria as that used in the 33 ms condition earlier.

We first inspected the dependence of the N170 on stimulus properties averaged across subjective confidence to replicate previous findings corroborating the graded hypothesis and then repeated the analysis only for high subjective confidence to exclude contamination from guessing. Moreover, we compared the “full” N170 for different durations in the same blocks to mitigate any effects of experimental context. Overall, our findings support the all-or-none hypothesis for the N170 generation.

In addition to the N170, we also analyzed the P1 in the same manner to inspect whether the effects of the N170 also occurred for other ERP components. The P1, which manifests as a positive voltage at posterior scalp electrodes, is mainly influenced by the low-level properties of stimuli, such as luminance, contrast, and spatial frequency. Critically, unlike the N170, the P1 does not respond specifically to faces (Rossion and Caharel 2011). We do not expect that the P1 would be modulated by the stimulus durations, as shown by Carbon et al. (2005). We further explored how the P1 amplitude was associated with the subjective confidence.

## Materials and methods

### Participants

We recruited 35 participants for this study. We instructed participants to press Key 1 only when they were sure that the stimulus was a face, with which the “full” N170 amplitudes for faces could be examined. Using an inclusion criterion of 95% correct in this condition, we excluded 7 participants (*M* = 76%, range: 49%—94%), who were not included in further analyses.

The remaining 28 participants (20 females, age range: 18–32 years, *M*_age_ = 22.32) all had normal or corrected-to-normal vision, participated in the experiment in exchange for course credits or supermarket vouchers, and gave written consent before completing the tasks. This study was approved by the University of Auckland Human Participants Ethics Committee (Reference Number 016833).

### Stimuli

**Fig. 1:**
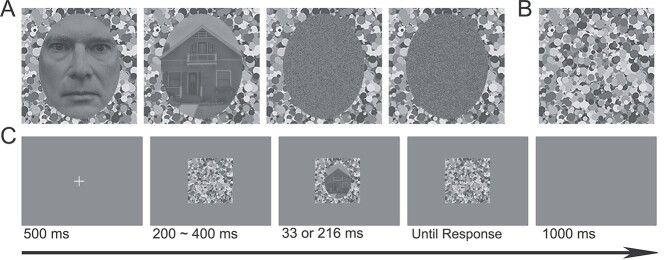
Stimuli and procedures in this experiment. A) Example face and house stimuli, as well as their corresponding scrambled versions, placed on the same masking background. Scrambled stimuli were created by randomizing the locations of all pixels within the oval contour. The face example image was not used in the experiment. B) The masking image. C) The trial procedure in this experiment.

The stimuli consisted of 40 photographs of Caucasian (20 female) faces from Ge et al. (2009) and 40 house images from Caltech Houses Database (Helle and Perona 2000). For face images, we removed all external features, such as ears, hair, and necks, by masking the image with an oval window (200 × 256 pixels, 4.94° × 6.32°). We applied the same oval window to the house images to ensure that all stimuli shared the same outline. In addition, we converted all the face and house images to grayscale and balanced their luminance using the SHINE toolbox (Willenbockel et al. 2010) in Matlab. We created a scrambled version of each image by randomizing the locations of all pixels of each image within the oval window using a custom Matlab script (available: https://osf.io/nxav8/). We show example face and house stimuli and their corresponding scrambled images in Fig. 1A. In addition to these stimuli, we created 4 face (2 female) and 4 house images for the practice trials. We displayed all stimuli against a 7° × 7° field of partially overlapping circles, which varied in size, location, and shade of gray (Fig. 1B). A mask that was identical in composition to this background appeared before and after stimulus presentation to disrupt any afterimage following the stimulus offset and minimize the influence of backward masking on the ERP evoked by experimental stimuli.

### Apparatus

We administered the experiments using E-prime 2.0 (Version 2.0.8.74, Psychology Software Tools, Pittsburgh, PA). Stimuli were displayed on a Samsung Sync Master HDTV monitor (P2270HD) with 1,920 × 1,080 resolution and a refresh rate of 60 Hz. The background color of the screen was gray (RGB values: 128, 128, 128). Participants were seated on a comfortable chair in a dimly lit, electrically shielded room (Belling Lee—Model L3000, Enfield, England). Responses were collected via the keyboard. The viewing distance between participants and the screen was maintained at about 57 cm.

Electroencephalography (EEG) was recorded continuously (1,000 Hz sample rate; 0.1–400 Hz analog bandpass, 400-MΩ input impedance) throughout each session using 128-channel Ag/AgCl electrode nets (Tucker 1993), the Geodesic EEG System 400 amplifier, and Net Station 4.5.7 from Electrical Geodesics Inc. (Eugene, OR). Electrode impedances were kept below 40 kΩ, an acceptable level for this system (Ferree et al. 2001). Common vertex (Cz) was used as a reference.

### Procedure

Each trial (Fig. 1C) in this study started with a fixation cross displayed for 500 ms. This was followed by a pre-stimulus mask with variable duration (200 ms, 250 ms, 300 ms, 350 ms, or 400 ms). Then the experimental stimulus was presented superimposed on the background for either 33 ms or 216 ms, (We submitted the parameters of 17 ms and 200 ms to E-prime as the stimulus durations but the actual durations were about 33 ms and 216 ms, respectively. These actual stimulus durations were calculated by the differences between the onsets of experimental stimuli and poststimulus masks recorded by the triggers sent to the raw EEG data.) followed by the poststimulus mask which remained visible until response. We asked participants to judge whether the stimulus was a face or a house by pressing 1 of 5 response keys. They were to press Key 1 if they were sure the stimulus was a face; Key 2 if they were not sure, but thought it was a face; Key 5 if they were sure it was a house; Key 4 if they were not sure, but thought it was a house; and Key 3 if they had no idea what it was. The interval between trials was 1,000 ms.

We employed a 2 (Type: intact vs. scrambled) × 2 (Category: faces vs. houses) × 2 (Duration: 33 ms vs. 216 ms) within-subject design in this study. There were 4 blocks in total, and each consisted of 320 trials. In the first 2 blocks, all stimuli were presented for 33 ms. Every intact stimulus was repeated 3 times, while every scrambled stimulus was displayed once in each of the first 2 blocks. In the last 2 blocks, every stimulus was presented once in each condition. In summary, each intact stimulus (40 faces and 40 houses) was presented 8 times in the 33 ms condition and twice in the 216 ms condition. Each scrambled stimulus (40 faces and 40 houses) was presented 4 times in the 33 ms and twice in the 216 ms condition. It took about 1 h to finish all these trials. In addition, prior to the main experiment, participants completed 32 practice trials, in which the practice stimuli were presented for 33 ms. No feedback was provided at the single-trial level, and the responses were summarized at the end of the practice.

### E‌EG data preprocessing

Preprocessing of EEG data was conducted using EEGLAB (Version 14.1.2, Delorme and Makeig 2004) and ERPLAB (Lopez-Calderon and Luck 2014) in Matlab (Version 9.3, MathWorks, Natick, MA). All the steps, except for rejecting the independent components based on the results of independent component analysis (ICA), were completed using Matlab scripts (available: https://osf.io/fvtc3/) in the High-Performance Computing cluster of New Zealand eScience Infrastructure.

The pre-processing pipeline mainly followed “Makoto’s suggestion” from Miyakoshi (2019). First, the options of EEGLAB were set to double precision. After loading the raw EEG data, the timestamps of all triggers were shifted 50 ms later to correct for amplifier and display delays. The amplifier delay was due to the amplifier taking time to process signals, which was reported by Electrical Geodesics Inc. (Eugene, OR) to be 36 ms for the Geodesic EEG System 400 amplifier with a 1,000 Hz sampling rate. The display delay was caused by the monitor taking time to display the stimuli. This delay time was measured by comparing the differences between the time point at which triggers of a stimulus were sent by E-prime and the time at which a photocell detected the change in luminance on the monitor, which was 14 ms on average. In total, the delay time was 50 ms. Then, the EEG data were downsampled from 1,000 Hz to 250 Hz and bandpass filtered (1 Hz–50 Hz). Next, the channel location information was loaded, and the line-noise frequencies at 50 Hz, 100 Hz, 150 Hz, 200 Hz, and 250 Hz were removed with the CleanLine (Version 1.04) plugin. Bad channels (4.11 channels per participant on average) were identified and removed with the clean_rawdata (Version 1.00) plugin and then interpolated. Next, the EEG data were re-referenced to the average of all the channels. ICA was conducted on the continuous EEG data with AMICA (Version 1.5.1), and the results were saved for further use. Note that a high-pass filter of 1 Hz was utilized in the initial part of the preprocessing analysis because high-pass filtered data at 1–2 Hz work better for ICA (Winkler et al. 2015). However, previous research also found that high-pass filtering (above 0.1 Hz) can distort the early ERP components (Acunzo et al. 2012). Therefore, we repeated all the above preprocessing steps except for the ICA analysis on the original data, but with a high-pass filter frequency of 0.1 Hz instead of 1 Hz. Then, the ICA results from the initial pass (filtered with high pass at 1 Hz) were applied to the data filtered with high pass at 0.1 Hz, as recommended by Miyakoshi (2019). The equivalent dipole source localization of independent components was calculated with dipfit (Version 3.0) plugin, which was followed by identifying the components representing bilateral source activities with fitTwoDipoles (Version 0.01) plugin (Piazza et al. 2016). We used the dipole information to reject components that did not appear to have a brain source. Next, the continuous EEG data were segmented into 1,500 ms epochs, starting 500 ms before the stimulus onsets. After completing all the above steps, we transferred the data to a local computer for further processing. Independent components identified as artifacts, such as eye blinks, horizontal eye movements, heartbeats, muscle contractions, and so on, were identified with SASICA (Chaumon et al. 2015), which implemented its own selection algorithms, and other 2 automated methods: ADJUST (Mognon et al. 2011) and FASTER (Nolan et al. 2010). Manually rejecting artefactual effects was mainly based on the results of SASICA and guided by Pion-Tonachini et al. (2019). After removing the artifact components, the data were reloaded to the cluster for further preprocessing. Improbable data were identified by joint probability and marked for all the channels. A linear detrend was applied from 200 ms prior to 996 ms after stimulus onset for each epoch. As the last step of preprocessing, the average voltage during the 200 ms prestimulus baseline was subtracted from each data point in each epoch.

### Integration windows, peak channels, and mean amplitudes

We used a data-driven method to identify appropriate integration windows for the N170 and P1 components that are unbiased by condition. To identify the N170 integration window, we chose P7 (E58) and P8 (E96) as the likely peak channels based on previous studies (Jemel et al. 2003; Rossion and Jacques 2008; Nemrodov et al. 2016). The cluster channels for P7 (E58) included electrodes E58, E65, E59, E51, E50, E57, and E64. The cluster channels for P8 (E96) included electrodes E96, E101, E97, E91, E96, E95, and E100. A grand-average waveform for the cluster of channels centered on P7 and P8 was obtained by averaging the data for the entire epoch across participants, conditions, and cluster channels. Then, we identified the minimum (i.e. most negative) amplitude of this grand-average waveform between 100 ms and 250 ms poststimulus. To compute the amplitude of the N170, we initially defined an integration window based on the full-width half-maximum of the component. If the width of this window was outside the range of 36–40 ms (a reasonable window for N170), the height–width ratio was adjusted iteratively until the integration window was within that range (inclusive).

After setting the N170 integration window, we created a grand mean scalp distribution for this period across all participants and conditions to allow visual confirmation of the peak channels. We observed that the centers of the negative peak activity were around PO7 (E65) and PO8 (E90), rather than our initial assumption of P7 and P8. Therefore, we updated the channel clusters to center on PO7 (channels E65, E70, E66, E59, E58, E64, and E69) and PO8 (E90, E96, E91, E84, E83, E89, and E95). We then rechecked the N170 integration window using the same procedure outlined above using the updated peak channels. As a result, the integration window for the N170 was defined as 152–192 ms (window size: 40 ms) poststimulus.

We used an analogous procedure to define the integration window for the P1 component, which we included in the analysis as a control, since we did not expect P1 to be modulated by perceptual categorization. We initially chose PO7 (E65) and PO8 (E90) as the likely scalp maxima of P1 based on previous literature (Kuefner et al. 2010; Rossion and Caharel 2011; Bankó et al. 2011; Smith 2012), and the maximum amplitude value of the grand-averaged epoch was identified between 70 ms and 140 ms after stimulus onsets. As a result, we set the integration window for P1 as 96 ms–136 ms (window size: 40 ms) poststimulus. The grand-average scalp distribution for this window confirmed that the peaks of P1 activity were at the PO7 and PO8 electrodes. Accordingly, the channel clusters for the P1 were identical to those identified for N170.

We defined the amplitudes for the 2 ERP components (P1 and N170) as the mean amplitudes within their respective integration windows. As a result, the latency of each component remained the same across different conditions. We measured the amplitudes in each window for every trial and then submitted the single-trial mean amplitudes to LMM for further analysis.

### Statistical analyses

#### Linear mixed modeling

We conducted LMM, instead of repeated-measure analysis of variance (rm-ANOVA), to assess single-trial mean amplitudes. There are several advantages of LMM, which make this technique popular as an alternative to rm-ANOVA (Baayen et al. 2002; Kristensen and Hansen 2004; Quené and van den Bergh 2004; Jaeger 2008; Aarts et al. 2014; Boisgontier and Cheval 2016). The specific benefit of applying LMM in this study is that, compared with rm-ANOVA, LMM can yield unbiased results in the presence of missing data without the need to remove participants (Kliegl et al. 2011; Linck and Cunnings 2015; Magezi 2015; Matuschek et al. 2017; Brauer and Curtin 2018). In this study, we expected that when intact face stimuli were presented for 33 ms, some participants may never perceive the stimuli with high subjective confidence and thus not use the Key 1 or Key 5 responses at all. We also thought it possible that some ratings may not be used by all participants. Another advantage of using LMM is that they can model amplitudes at the level of single observations and estimate multiple random effects. Estimating both participant and stimulus random effects maximizes data use and allows generalization of fixed effects to other similar participants and stimuli.

The general procedures to obtain an optimal model for each dependent variable followed the recommendations of Bates, Kliegl, et al. (2015a) and Matuschek et al. (2017). First, the maximal model was created, in which the random effect structure included all by-participant and by-item random intercepts and random slopes. If the maximal model failed to converge, the corresponding zero-correlation parameter model, in which the correlations between random effects were forced to be zero, was fitted. Then, the random effects that did not significantly explain the data were removed from the zero-correlation parameter model, resulting in the reduced model. The extended model was then built by adding back the correlations between the remaining random effects in the reduced model. If the extended model still could not converge, the random effects with the smallest amount of variance were removed one-by-one until a converged model was achieved, resulting in the final extended model. Next, the reduced and extended models were compared, and the one that explained data better (with smaller Akaike’s Information Criterion; Akaike 1998) was defined as the optimal model. Follow-up comparisons were conducted based on this optimal model.

In this study, we were interested in both the overall ERPs and the ERP differences between faces and nonface objects. First, to inspect how P1 and N170 amplitudes varied when intact faces were presented for different durations and were the object of distinct subjective confidence, we performed pairwise contrasts for all Duration and Confidence levels separately on the results of the optimal LMM for intact faces. Second, to test the dependency of the aspect of the ERP that could be specifically attributed to face processing (here defined as the difference between the face-related and house-related P1 and N170 amplitudes) for intact stimuli on durations and subjective confidence, we computed “pairwise interaction contrasts” between Category and Duration (or Confidence) for intact faces (i.e. simple interaction analysis; Jin 2021). For instance, the pairwise interaction contrast between Category and Duration in this study is equivalent to (33 ms faces – 33 ms houses) – (216 ms faces – 216 ms houses), i.e. the differences of face-minus-house ERP component between durations.

#### *Null hypothesis significance tests and* ET*s*

NHST remains one of the most popular inferential statistical methods used by researchers. When the probability (i.e. *P*-value) of observing the data or more extreme cases under the null hypothesis is small (less than some pre-defined α level), we reject the null hypothesis and infer that the alternative hypothesis is true. However, when that probability is not small enough, we cannot conclude whether the null hypothesis is true or whether there is insufficient evidence to make any decision (Cohen 1994).

In this study, we expected some null effects. For example, comparable N170 amplitudes may be generated for faces presented for different durations when they are perceived with high subjective confidence. Such effects can never be positively supported by conventional NHST. Therefore, in addition to conventional NHST, we employed ETs in this study to test whether an effect was equivalent to a region of null effects (Rogers et al. 1993; Seaman and Serlin 1998; Mara and Cribbie 2012; Meyners 2012; Weber and Popova 2012; Lakens 2017; Lakens et al. 2018). ET essentially performs 2 one-sided tests to determine whether the observed effects are outside a region that is believed to be equivalent to a null effect. In other words, the null hypothesis being tested in ET is that the observed effect is smaller than the lower boundary of the equivalence region or larger than its upper boundary. In this study, we defined this region of practical equivalence to zero as [−0.5 μV, 0.5 μV], which was reasonably small considering that ERP data are usually noisy. If the probability that the observed effects being outside the region was low (i.e. *P*-values for the 2 one-sided tests are both smaller than the conventional α level of 0.05), we rejected that hypothesis and inferred that the observed effect was statistically equivalent to the null effect. If the probability of any direction was not small enough, we could not infer the effect was equivalent to the null effect.

**Fig. 2:**
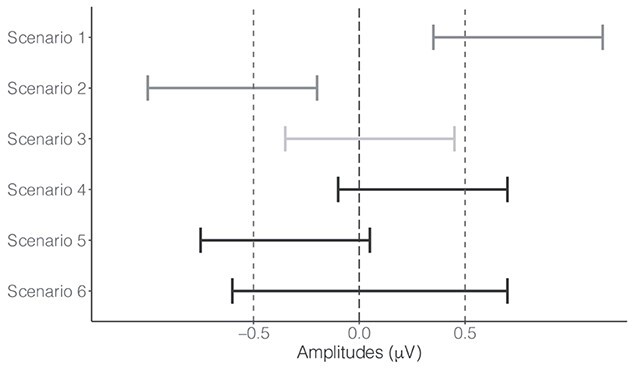
Scenarios of results obtained from conventional NHST and ET using hypothetical data. The black long-dashed vertical line denotes the effect of zero, which represents the null hypothesis in NHST. The gray dashed vertical lines denote the lower and upper boundaries [−0.5, 0.5] of the region equivalent to the null effect in ET. The horizontal error bars denote the confidence intervals of different effects. Scenarios 1 and 2 denote that the NHST is significant but the ET is not, which provides the evidence that the effect of interest is different from zero. Scenario 3 denotes that the ET is significant but the NHST is not, which provides the evidence that the effect of interest is equivalent to the null effect. Scenarios 4–6 denote that the effects are inconclusive with the evidence from NHST and ET.

In summary, we employed both conventional NHST and ET in this study to examine whether an effect was significantly different from zero and whether it was practically equivalent to the region of null effects, respectively. There are (at least) 6 possible scenarios that can be obtained by combining conventional NHST and ET (see Fig. 2 for a schematic representation of these possibilities). Scenarios 1 and 2 reflect instances in which the null hypothesis is rejected. In these instances, we only reported the results of NHST. Scenario 3 reflects cases in which there is evidence in favor of the null effects. Here, we only reported the results of ET. Since 2 one-sided tests are performed in ET, we only reported the results of the one-sided test whose absolute *t*-value was smaller and thus *P*-value is larger (Lakens et al. 2018). For the other scenarios, in which the null hypothesis can neither be rejected nor supported, and, therefore, the effects are inconclusive, we reported results of both NHST and ET.

#### Software

We performed all statistical analyses using R (R Core Team 2021) and RStudio (RStudio Team 2021). Data were tidied up with the Tidyverse package (Wickham et al. 2019). We performed LMM using the lme4 package (Bates, Mächler, et al. 2015b) and follow-up comparisons and ET with the emmeans package (Lenth 2021) where Satterthwaite approximation was used to estimate the statistical results. The R Markdown output file is available at https://osf.io/zv6kg/.

## Results

### Allocation of keypress responses

**Fig. 3:**
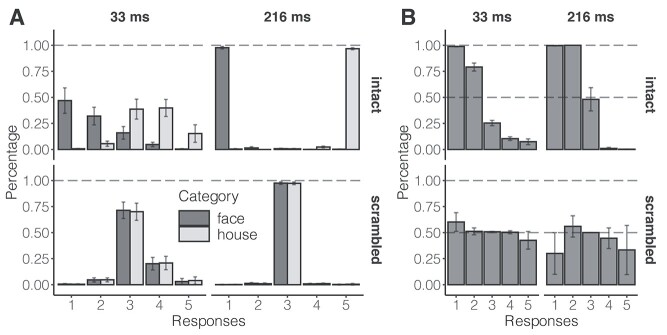
Allocation of keypress responses. Response 1 denotes participants were sure the stimulus was a face; response 2 denotes participants were not sure but thought it was a face; response 3 denotes participants had no idea what it was; response 4 denotes participants were not sure but thought it was a house; and response 5 denotes participants were sure it was a house. Error bars represent 95% CIs. A) The averaged responses within each condition. B) The percentage for each response and condition of trials in which the stimulus was a(n) (intact or scrambled) face. For instance, the leftmost column in the first row of panel (B) shows that almost all intact stimuli were faces when participants responded with Key 1.

The allocation of responses in each condition is summarized in Fig. 3A. Inspection of this figure reveals that, for brief (33 ms) presentations, faces were perceived more frequently and with higher confidence than houses. Specifically, when intact faces were presented, participants reported being sure that they had seen a face (Key 1) on about half of trials (*M* = 0.469, 95% confidence interval (CI): [0.346, 0.591]), and unsure, but thought the stimulus was a face (key 2) on a further third of the trials (*M* = 0.321, 95% CI: [0.236, 0.405]). When intact houses were presented, participants were less confident. The most common responses were that they were unsure, but thought the stimulus was a house (Key 4; *M* = 0.399, 95% CI: [0.317, 0.481]), had no idea what were presented (Key 3; *M* = 0.387, 95% CI: [0.292, 0.483]), and were sure the stimulus was a house (Key 5; *M* = 0.153, 95% CI: [0.069, 0.238]). When the corresponding scrambled images were presented for 33 ms, participants most commonly responded that they had no idea what stimulus was presented (Key 3; scrambled faces: *M* = 0.714, 95% CI: [0.635, 0.793]; scrambled houses: *M* = 0.700, 95% CI: [0.618, 0.782]). As expected, when the stimuli were presented for 216 ms, participants were essentially perfect in discriminating intact faces from intact houses and had high confidence in their responses (Key 1; faces: *M* = 0.977, 95% CI: [0.965, 0.989]; houses: *M* = 0.967, 95% CI: [0.957, 0.977]). When the images were scrambled, the participants once again consistently reported having no idea whether they depicted faces or houses (Key 3; faces: *M* = 0.975, 95% CI: [0.963, 0.988]; houses: *M* = 0.973, 95% CI: [0.960, 0.986]).

Figure 3B depicts the percentage of stimuli being (intact or scrambled) faces for each response in the intact and scrambled conditions. For instance, the leftmost column in the first row depicts the percentage of stimuli being intact faces when participants responded with Key 1 for intact stimuli presented for 33 ms. If participants were following instructions appropriately, they should have pressed Keys 1 and 5 only when they were completely sure of the stimulus. Note that we removed participants who failed to reach 95% accuracy for Key 1 before conducting any further analysis (see Introduction and Participant). Indeed, regardless of stimulus duration, when participants responded with Key 1, almost all the stimuli were intact faces (33 ms: *M* = 0.988, 95% CI = [0.983, 0.994]; 216 ms: *M* = 0.996, 95% CI = [0.993, 0.999]), while only very few stimuli were faces when participants responded with Key 5 (33 ms: *M* = 0.074, 95% CI = [0.020, 0.128]; 216 ms: *M* = 0.002, 95% CI = [0.000, 0.004]). In addition, we also instructed participants to select Key 3 when they were unable to categorize the stimuli. For scrambled stimuli, this resulted in chance performance for Key 3 responses (33 ms: *M* = 0.508, 95% CI = [0.500, 0.515]; 216 ms: *M* = 0.501, 95% CI = [0.499, 0.502]).

These data confirm that participants were using the subjective confidence scale appropriately and distributing their responses across the 5 response options. Critically, the variability in subjective confidence when the stimuli were presented for 33 ms enabled us to explore further the associations between subjective confidence and ERP amplitudes.

### ERP amplitudes averaged across responses

**Fig. 4:**
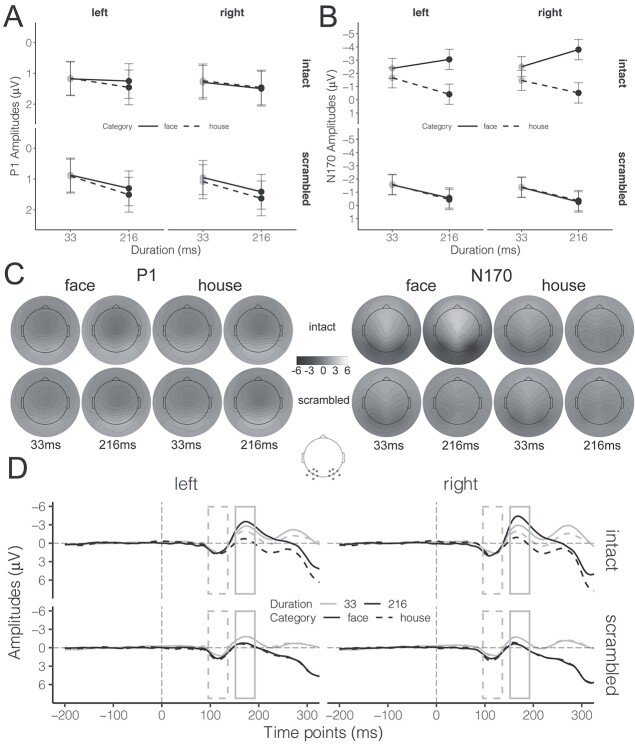
ERP results as a function of Hemisphere (left vs. right), Type (intact vs. scrambled), Category (faces vs. houses), and Duration (33 ms vs. 216 ms) across behavior responses. A and B) The estimated marginal means of P1 and N170 amplitudes, respectively; error bars represent 95% CIs. C) Topographic maps corresponding to the P1 (left panel) and N170 (right panel) components. The gray dots in the schematic brain denote the channel locations for both P1 and N170 on both hemispheres. D) The grand-average ERP waveforms. The gray dashed rectangle denotes the integration window for the P1 (96 ms–136 ms), while the gray solid rectangle denotes the integration windows for the N170 (152 ms–192 ms). Please see Supplementary Fig. S1 for color topographic maps.

We used LMM to analyze the amplitudes of the P1 and N170 components of the ERP in 3 different ways. First, we collapsed across response options to replicate the effects of stimulus duration reported in previous literature (Carbon et al. 2005; Tanskanen et al. 2007; Mitsudo et al. 2011), in which N170 amplitudes were larger for longer durations compared with shorter durations. While our main focus was on modulation of N170, we also analyzed P1 amplitudes (which we did not expect to be modulated by face stimuli; Rossion and Caharel 2011) to account for any effects that result from general amplification of the ERP as a function of stimulus duration. The grand-average ERP waveforms and topographic maps corresponding to the P1 and N170 integration windows are shown in Fig. 4D and C, respectively. We fitted amplitudes for each component using LMM and the results were submitted to pairwise contrasts and pairwise interaction contrasts to test the influence of stimulus properties on P1 and N170 amplitudes, as well as on the face-minus-house ERP component.

#### P1 amplitudes

The estimated marginal means of P1 amplitude for each condition are depicted in Fig. 4A. To examine the dependency of P1 amplitudes on stimulus properties, we performed pairwise contrasts corrected by the Bonferroni methods for 2 tests. Results revealed that the differences in P1 amplitudes evoked by intact faces between 33 ms and 216 ms conditions were statistically equivalent to the null effect for the left-hemisphere electrodes (*t*(123.0) = 2.61, *P* = 0.01, *b* = −0.07, 90% CI = [−0.40, 0.25]), but remained inconclusive for the right-hemisphere electrodes (NHST: *t*(123.0) = −1.24, *P* = 0.44; ET: *t*(123.0) = 1.80, *P* = 0.07, *b* = −0.20, 90% CI = [−0.53, 0.12]). Moreover, to examine the dependency of P1 amplitude differences between faces and houses on stimulus properties, we performed pairwise interaction contrasts between Category and Duration for intact stimuli, with Bonferroni corrections for 2 tests. No significant face-related modulation of the P1 component by stimulus duration was found for the right-hemisphere electrodes (*t*(266.2) = −3.12, *P* = 0.002, *b* = 0.01, 90% CI = [−0.29, 0.32]), whereas this modulation was inconclusive for the left-hemisphere electrodes (NHST: *t*(266.2) = 1.44, *P* = 0.30; ET: *t*(266.2) = −1.76, *P* = 0.08, *b* = 0.22, 90% CI = [−0.08, 0.53]). Both analyses suggest that neither the overall P1 nor the face-minus-house P1 components were affected by durations, implying that any differences found for N170 amplitudes later could not be attributed to generalized amplification of the ERP for longer stimulus duration.

#### N170 amplitudes

The estimated marginal means of N170 amplitudes in each condition are displayed in Fig. 4B. In general, longer stimulus duration was associated with a weaker (lower amplitude) N170, except when the stimuli were intact faces. Pairwise contrasts, with Bonferroni corrections for 2 tests, confirm that the N170 amplitudes evoked by intact faces were weaker for 33 ms relative to 216 ms presentations for both hemisphere electrodes (left: *t*(100.1) = 2.62, *P* = 0.02, *b* = 0.68, 95% CI = [0.09, 1.26]; right: *t*(100.1) = 5.02, *P* < 0.001, *b* = 1.30, 95% CI = [0.71, 1.88]). In other words, longer exposure to faces resulted in larger N170 amplitude, whereas in all other conditions, longer exposure resulted in lower amplitude.

Next, we compared the face-minus-house N170 components for different durations via pairwise interaction contrasts between Category and Duration for intact stimuli. These contrasts revealed significant interactions between Category and Duration for both hemisphere electrodes (left: *t*(104.1) = 8.93, *P* < 0.001, *b* = 1.92, 95% CI = [1.43, 2.41]; right: *t*(104.1) = 10.40, *P* < 0.001, *b* = 2.24, 95% CI = [1.75, 2.73]), suggesting that N170 amplitude differences between faces and houses were stronger for 216 ms than 33 ms conditions.

These results seem to support the view that the N170 for faces and its face-minus-house component are both graded. However, we will return to this issue in subsequent analyses.

### Associations between subjective confidence and ERP amplitudes

The previous analyses compared P1 and N170 amplitudes for face and house images—as well as scrambled versions of both—regardless of the participants’ responses. While the results were consistent with the idea that the N170 (but not the P1) is graded in amplitude as a function of the amount of face information, it is possible that the N170 was contaminated by including trials in which no face was perceived. This would be expected to reduce the N170 for the short-duration (33 ms) condition more than the long-duration (216 ms) condition, perhaps creating an illusion of a graded response. Accordingly, we repeated the analysis taking subjective confidence into account. As shown in Fig. 3A, we mostly observed variability in response when intact stimuli were presented for 33 ms. In particular, most responses for 33 ms intact faces were Key 1, Key 2, and Key 3, whereas most responses for 33 ms intact houses were Key 5, Key 4, and Key 3. These keys corresponded to high confidence, low confidence, and guessing for face and house stimuli, respectively. Thus, we converted these responses into a new factor, Confidence, which had 3 levels: high (Key 1 for faces or Key 5 for houses), low (Key 2 for faces or Key 4 for houses), and guess (Key 3 for both stimuli). Since we also wished to include the 216 ms intact stimuli conditions as the baseline level, and most responses in these conditions were Key 1 and Key 5 (i.e. high confidence for faces and houses respectively), we further integrated Confidence with Duration to create a new factor, Duration Confidence, with 4 levels: 33 ms_guess, 33 ms_low, 33 ms_high, and 216 ms_high. We did not include scrambled trials in the analyses.

**Fig. 5:**
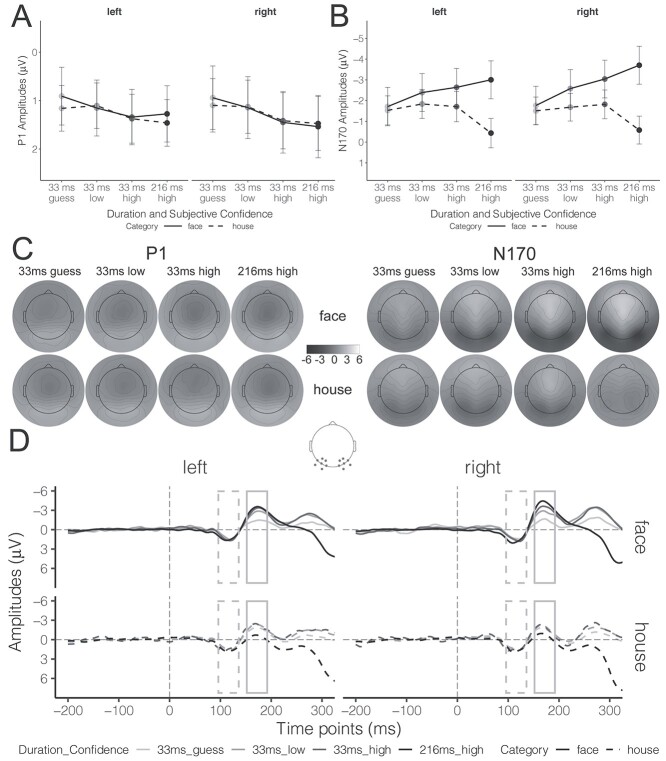
ERP results for intact stimuli as a function of Hemisphere (left vs. right), Category (faces vs. houses), and Duration Confidence (33ms_guess, 33 ms_low, 33 ms_high, vs. 216 ms_high). A and B) The estimated marginal means of P1 and N170 amplitudes, respectively; error bars represent 95% CIs. C) Topographic maps corresponding to the P1 and N170 components. The gray dots in the schematic brain denote the channel locations for both P1 and N170 on both hemispheres. D) The grand-average ERP waveforms. The gray dashed rectangle denotes the integration window for the P1 (96 ms–136 ms), while the gray solid rectangle denotes the integration windows for the N170 (152 ms–192 ms). Please see Supplementary Fig. S2 for color topographic maps.

The grand-average ERP waveforms and topographic maps corresponding to the P1 and N170 integration windows are shown in Fig. 5C (topographies) and Fig. 5D (waveforms). We analyzed the LMM results using pairwise contrasts and pairwise interaction contrasts to test the influence of Duration Confidence on overall ERP amplitudes and the face-minus-house ERP component.

#### P1 amplitudes

The estimated marginal means of P1 amplitude for each condition are depicted in Fig. 5A. Similar to the earlier analyses, we performed pairwise contrasts to test the dependency of P1 amplitudes on Duration Confidence, with Bonferroni corrections for 6 tests, and the detailed statistical results are available in Supplementary Table S1. For the left-hemisphere electrodes, P1 amplitudes in 33 ms_guess conditions were weaker than those in 33 ms_high conditions (*t*(20687.5) = −3.14, *P* = 0.01, *b* = −0.43, 95% CI = [−0.80, −0.07]). Differences between 33_low, 33_high, and 216_high were statistically equivalent to the null effect (|*t*| > 2.86, *P* < 0.02) while the comparisons between 33 ms_guess and 33 ms_low, as well as between 33 ms_guess and 216 ms_high, were not conclusive (NHST: |*t*| < 2.57, *P* > 0.06; ET: |*t*| < 1.89, *P* > 0.18). For the right-hemisphere electrodes, P1 differences between 33 ms_high and 216_high were statistically equivalent to the null effect (*t*(34348.2) = 3.76, *P* < 0.001, *b* = −0.09, 90% CI = [−0.35, 0.17]). Moreover, P1 amplitudes for 33 ms_guess and 33 ms_low were weaker than those for 33 ms_high and 216 ms_high (|*t*| > 2.79, *P* < 0.04). And P1 differences between 33 ms_guess and 33 ms_low were not conclusive (NHST: *t*(35346.6) = −1.51, *P* = 0.79; ET: *t*(35346.6) = 2.19, *P* = 0.09). These results indicate that, in general, weaker P1 amplitudes were associated with lower subjective confidence but the effects were quite small, usually smaller than 1 μV.

We also assessed face-minus-house P1 amplitudes with pairwise interaction contrasts between Category and Duration Confidence, which were corrected with Bonferroni methods for 6 tests, and the detailed statistical results are available in Supplementary Table S1. For the left-hemisphere electrodes, we found equivalence to null effects only for the comparisons between 33 ms_guess and 216 ms_high (*t*(31977.1) = 2.42, *P* = 0.05, *b* = −0.06, 90% CI = [−0.50, 0.37]) and between 33 ms_low and 33 ms_high (*t*(7492.4) = −2.43, *P* = 0.05, *b* = 0.08, 90% CI = [−0.33, 0.49]); the other comparisons remained inconclusive (NHST: |*t*| < 1.79, *P* > 0.44; ET: |*t*| < 1.92, *P* > 0.16). For the right-hemisphere electrodes, we found that the differences in face-minus-house P1 amplitudes between the 33 ms_low, 33 ms_high, and 216 ms_high were statistically comparable (|*t*| > 2.58, *P* < 0.03), while other comparisons were inconclusive (NHST: |*t*| < 1.23, *P* > 0.99; ET: |*t*| < 1.94, *P* > 0.15). In comparison to the overall P1 amplitude results, the face-minus-house P1 component was less likely to be influenced by subjective confidence. That is, variations in P1 amplitude as a function of subjective confidence cannot be attributed to modulation of a face-specific response but are instead likely to reflect general visual processing.

#### N170 amplitudes

The estimated marginal means of N170 amplitudes in each condition are displayed in Fig. 5B. We conducted pairwise contrasts to compare the differences in N170 amplitudes evoked by intact faces between levels of Duration Confidence, with Bonferroni corrections for 6 tests, and the statistical results are available in Supplementary Table S1. For the left-hemisphere electrodes, N170 amplitudes for 33 ms_guess were weaker than all other levels of Duration Confidence (|*t*| > 4.84, *P* < 0.001). Differences of N170 amplitudes between 33 ms_low and 33 ms_high conditions were not conclusive (NHST: *t*(27206.8) = 2.15, *P* = 0.19; ET: *t*(27206.8) = −2.12, *P* = 0.10) and the amplitudes elicited in both of these conditions were weaker than those in the 216 ms_high condition (|*t*| > 3.20, *P* < 0.009). A similar pattern occurred for the right-hemisphere electrodes, with all differences reaching significance. Specifically, N170 amplitudes for 33 ms_guess were weaker than the other 3 levels (|*t*| > 5.85, *P* < 0.001). N170 amplitudes for 33 ms_low were weaker than 33 ms_high and 216 ms_high (|*t*| > 3.95, *P* < 0.001). Critically, when the subjective confidence was high, N170 amplitudes for the 33 ms conditions were still weaker than for 216 ms (*t*(36540.1) = 5.88, *P* < 0.001, *b* = 0.66, 95% CI = [0.37, 0.96]). These results revealed that stronger (absolute) N170 amplitudes were associated with higher subjective confidence. Notably, even when perceived with similarly high subjective confidence, faces presented for 216 ms still evoked stronger N170 amplitude compared to 33 ms.

We examined the face-minus-house N170 component using pairwise interaction contrasts between levels of Category for each level of Duration Confidence, with Bonferroni corrections for 6 tests, and the statistical results are available in Supplementary Table S1. For the left-hemisphere electrodes, face-minus-house component in the 216 ms_high condition was stronger than all other 3 levels (|*t*| > 8.52, *P* < 0.001). N170 amplitude in the 33 ms_high condition was also stronger than in 33 ms_guess condition (*t*(8673.1) = −3.52, *P* = 0.003, *b* = −0.75, 95% CI = [−1.31, −0.19]). Differences between other levels were inconclusive (NHST: |*t*| < 2.10, *P* > 0.21; ET: |*t*| < 0.76, *P* > 0.99). For right-hemisphere electrodes, the face-minus-house components differed between all levels except for the comparison between 33 ms_low and 33 ms_high, which remained inconclusive (NHST: *t*(5675.1) = −1.75, *P* = 0.49; ET: *t*(5675.1) = −0.96, *P* > 0.99). Specifically, the N170 was stronger in the 216 ms_high condition than the other 3 levels (|*t*| > 10.01, *P* < 0.001). The N170 amplitudes in the 33 ms_high and 33 ms_low conditions were stronger than those in the 33 ms_guess condition (|*t*| > 3.66, *P* < 0.002). Similar to the results of N170 amplitudes, stronger face-minus-house N170 component was also associated with higher subjective confidence.

### Amplitudes from trials with high confidence

The analyses conducted so far confirmed that both the overall N170 and the face-minus-house N170 component appeared to be graded with stimulus duration and subjective confidence that the stimulus was a face. However, a conclusion that the N170 was generated in a graded fashion would be premature. In the first half of the experiment, we presented all stimuli for 33 ms, while in the second half the stimulus duration was either 33 ms or 216 ms. It remained possible that, although the N170 was generated all-or-none, this discrepancy in parameters might result in distinct amplitudes of the “full” N170 in the 2 halves, which further led to the observed differences of N170 between 33 ms and 216 ms conditions in the above results. In other words, if the “full” N170 differed between the 2 halves, the conclusion that the N170 was generated in a graded manner would be undermined. In this case, the generation of the N170 needed to be tested with faces presented for different durations in the same blocks where the “full” N170 remained constant. On the contrary, if the “full” N170 was similar for the 2 halves, it would be safer to draw the conclusion that the N170 was graded. Therefore, it was critical to examine whether the “full” N170 was different between the first and second halves before drawing a conclusion about the nature of the N170 generation. To do so, we compared N170 amplitudes for faces that were presented for 33 ms and perceived with high subjective confidence in the 2 halves to explore whether the “full” N170 for these conditions was comparable. We additionally inspected the amplitudes for faces presented for 33 ms and 216 ms with high subjective confidence in the same half to examine whether the N170 was generated in graded fashion or was all-or-none in nature. Because we only included the 216 ms duration in the second half of the experiment, we created a new factor for the LMM analysis, Duration Half, which had 3 levels: 33 ms_half1, 33 ms_half2, and 216 ms_half2.

**Fig. 6:**
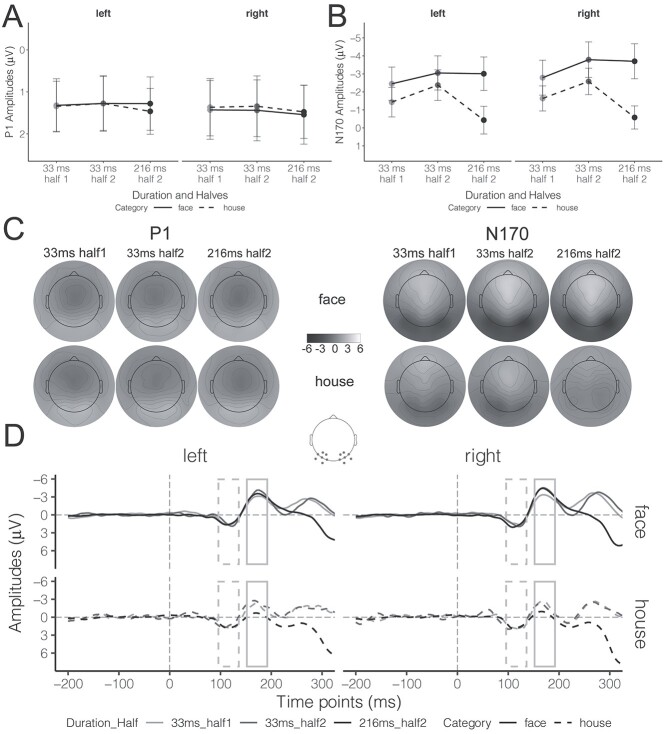
ERP results for intact stimuli with high subjective confidence as a function of Hemisphere (left vs. right), Category (faces vs. houses), and Duration Half (33ms_half1, 33 ms_half2, vs. 216 ms_half2). A and B) The estimated marginal means of P1 and N170 amplitudes, respectively; error bars represent 95% CIs. C) Topographic maps corresponding to the P1 and N170 components. The gray dots in the schematic brain denote the channel locations for both P1 and N170 on both hemispheres. D) The grand-average ERP waveforms. The gray dashed rectangle denotes the integration window for the P1 (96 ms–136 ms), while the gray solid rectangle denotes the integration windows for the N170 (152 ms–192 ms). Please see Supplementary Fig. S3 for color topographic maps.

The grand-average ERP waveforms and topographic maps corresponding to the P1 and N170 integration windows are shown in Fig. 6D and C, respectively. We submitted the LMM results to pairwise contrasts and pairwise interaction contrasts to test the influence of Duration Half on overall ERP amplitudes and the face-minus-house components of both P1 and N170.

#### P1 amplitudes

The estimated marginal means of P1 amplitudes in each condition are displayed in Fig. 6A. Pairwise contrasts, with Bonferroni corrections for 3 tests, explored the influence of Duration Half on P1 amplitude. These contrasts reveal that all comparisons were equivalent to the null effect for both hemisphere electrodes (|*t*| > 2.49, *P* < 0.007). Pairwise interaction contrasts for the face-minus-house P1 component, again with Bonferroni corrections for 3 tests, revealed inconclusive results (NHST: |*t*| < 0.76, *P* > 0.99; ET: |*t*| < 1.71, *P* > 0.13) with the exception that the comparison between 33 ms_half1 and 216 ms_half2 was equivalent to the null effect (*t*(4863.0) = 2.30, *P* = 0.03, *b* = −0.00, 90% CI = [−0.46, 0.46]). Overall, these results revealed no systematic influence of Duration Half on P1 amplitudes for intact stimuli perceived with high confidence.

#### N170 amplitudes

The estimated marginal means of N170 amplitudes in each condition are displayed in Fig. 6B. Differences of N170 amplitudes between levels of Duration Half were compared with pairwise contrasts, using Bonferroni corrections for 3 tests. These contrasts revealed 2 important results, which were similar for both hemispheres. First, N170 amplitudes were weaker for the 33 ms_half1 condition compared to those for both the 33 ms_half2 (left: *t*(18306.7) = 3.97, *P* < 0.001, *b* = 0.60, 95% CI = [0.24, 0.96]; right: *t*(18304.0) = 6.59, *P* < 0.001, *b* = 1.00, 95% CI = [0.64, 1.36]) and 216 ms_half2 (left: *t*(17686.7) = 4.38, *P* < 0.001, *b* = 0.56, 95% CI = [0.25, 0.86]; right: *t*(17334.1) = 7.17, *P* < 0.001, *b* = 0.91, 95% CI = [0.61, 1.22]) conditions. These results, especially the differences between 33 ms_half1 and 33 ms_half2, suggest that the “full” N170 differed between the 2 halves of the experiment. Thus, it was critical to inspect the N170 generation with amplitudes in the same blocks, as shown following. In the second half of the experiment (i.e. the same blocks), the differences in N170 amplitudes between 33 ms and 216 ms conditions were statistically equivalent to the null effect (left: *t*(18181.2) = 2.79, *P* = 0.008, *b* = −0.04, 90% CI = [−0.39, 0.30]; right: *t*(18079.2) = 2.53, *P* = 0.02, *b* = −0.09, 90% CI = [−0.43, 0.26]). This result suggests that a “full” N170 is generated once a face is perceived with high subjective confidence regardless of stimulus duration.

We also analyzed the influence of Duration Half on the face-minus-house N170 component, again using pairwise interaction contrasts corrected with Bonferroni methods for 3 tests. We again found similar effects for both hemispheres. The differences in face-minus-house N170 component between 33 ms_half1 and 33 ms_half2 conditions were inconclusive for both hemispheres (NHST: |*t*| < 1.3, *P* > 0.68; ET: |*t*| < 1.56, *P* > 0.17). Nevertheless, the amplitudes in both conditions were weaker than those for the 216 ms_half2 condition (33ms_half1 left hemisphere: *t*(7112.0) = 6.58, *P* < 0.001, *b* = 1.56, 95% CI = [0.99, 2.12]; 33 ms_half1 right hemisphere: *t*(2326.2) = 8.58, *P* < 0.001, *b* = 1.97, 95% CI = [1.42, 2.52]; 33 ms_half2 left: *t*(13731.6) = 6.68, *P* < 0.001, *b* = 1.90, 95% CI = [1.22, 2.58]; 33 ms_half2 right: *t*(7615.6) = 6.82, *P* < 0.001, *b* = 1.92, 95% CI = [1.24, 2.59]). The face-minus-house N170 component was stronger when faces were presented for 216 ms relative to 33 ms, although stimuli were perceived with high subjective confidence in each case.

## Discussion

We sought a resolution to the question of whether visual categorization is supported by graded or all-or-none neural mechanisms by inspecting the generation of the N170. To achieve this, we explored the influence of stimulus category (face vs. house; intact vs. scrambled) and duration (33 ms vs. 216 ms) on the N170 amplitudes, as well as their associations with subjective confidence. We analyzed these components in 3 different ways. In our first analysis, in which we averaged across subjective confidence, we replicated previous observations (Carbon et al. 2005; Tanskanen et al. 2007) that N170 amplitudes evoked by intact faces were greater for longer compared to shorter stimulus durations. This finding also concurred with earlier observations of the effects of stimulus onset asynchrony (Nasr 2010; Eiserbeck et al. 2021) and visual noise (Jemel et al. 2003; Philiastides et al. 2006; Philiastides and Sajda 2006; Schneider et al. 2007; Bankó et al. 2011; Navajas et al. 2013) on N170 amplitudes. These studies, and the results of our first analysis, suggest that stimuli with more facial information (temporal or spatial) elicit stronger N170 (or M170) responses. In other words, without considering subjective confidence, our first analysis appears to support the hypothesis that the N170 is a graded response. However, neither our first analysis nor those presented in the earlier studies can rule out the possibility that the N170 amplitudes observed for brief stimuli were lower because the response was diluted by including trials in which the face stimulus was not detected.

Since detected faces usually evoked greater N170 responses than undetected faces (Fisch et al. 2009; Rodríguez et al. 2012; Navajas et al. 2013), our second analysis explored N170 amplitudes further by considering subjective confidence. In the critical conditions, in which stimuli were correctly categorized with equally high subjective confidence, faces presented for longer durations still evoked greater N170 amplitudes. This finding also appears to support the hypothesis that N170 is a graded response that reflects the subjective strength of the percept of a face. However, this conclusion, too, was undermined because the discrepancy in parameters might lead to differences in the “full” N170 between the first and second halves of the experiment, which in turn could explain the different N170 amplitudes for varying durations. Accordingly, we further examined differences between the N170 evoked by briefly presented faces in the 2 halves, as well as between faces presented for 33 ms or 216 ms in the second half of the recording session.

This final analysis revealed, first, that N170 amplitudes evoked by faces presented for 33 ms and perceived with high subjective confidence were stronger in the second half of the experiment than the first. This might stem from the practice effect and/or lack of previous knowledge of the faces during the first half where all stimuli were presented for 33 ms. Participants might not have had a clear idea of what the face images looked like until they saw the longer-duration stimuli in the second half. In spite of the fact that N170 amplitudes evoked by intact faces for 33 ms were stronger in the second half of the experiment, the face-minus-house component was more likely to remain comparable between the 2 halves. Hence, the differences of the N170 between halves should be attributable to the nonface contribution to the N1/N170 complex. This implies that no matter what caused the stronger N170 in the second halves, the influence on the N170 was not specific to faces.

Since the “full” N170 differed between the first and second halves of the experiment, the N170 generation needed to be tested within the same blocks. ET revealed that N170 amplitudes in the second half were comparable between 33 ms and 216 ms conditions when faces were perceived with high subjective confidence. This result suggests that rather than being a graded response, the N170 is generated in an all-or-none manner once a face is perceived. All stimuli that were correctly perceived as faces with high subjective confidence appear to have evoked similar N170 amplitudes, regardless of stimulus duration. This result also implies that the appearance that N170 grades in strength with stimulus information or subjective confidence is an artifact of mixing heterogeneous trial types in signal averaging.

Distinct from the comparable N170 amplitudes in the 33 ms and 216 ms conditions with high subjective confidence, the face-minus-house N170 component was higher in amplitude in the 216 ms condition than in the 33 ms condition when similar high subjective confidence was reported. This result does not seem to be consistent with a recent study which employed rapid serial visual presentation and did not observe the differences in face-minus-nonface signals evoked by stimuli presented for different durations (Retter et al. 2020). This inconsistency may result from the different methods used in the 2 studies, and therefore, the signals measured may reflect different aspects of ERPs. More specifically, Retter et al. (2020) analyzed the implicit frequency-tagged face-minus-nonface EEG responses, which were likely to reflect the combined contributions of both early and late ERP components. In other words, their results may suggest that the mixed combinations of face-minus-nonface P1, N170, N250, and other later components were comparable when faces were perceived regardless of stimulus duration. By contrast, our study measured the ERPs for each component directly. These results suggest that the face-minus-house N170 component was stronger for stimuli presented for longer durations. This finding, in addition to the observation of comparable N170 for faces with varying durations, indicates that although stimulus duration impacted the general component, potentially also the face-minus-house N1/N170 component, a “full” N170 is always achieved whenever a face is perceived, corroborating that the N170 is generated in all-or-none.

In addition to the N170, we also examined P1 amplitudes in each condition. We found similar P1 amplitudes evoked by intact stimuli in 33 ms and 216 ms conditions, consistent with Carbon et al. (2005). However, some previous studies have reported that faces embedded in noise evoked stronger P1 amplitudes (Bankó et al. 2011; Rousselet et al. 2008; Schneider et al. 2007; Tanskanen et al. 2005; Tarkiainen et al. 2002; but see Jemel et al. 2003). The discrepancy might result from the fact that embedding faces in noise altered the low-level properties of stimuli, as well as the facial information. In contrast, the low-level information of stimuli remained the same when they were presented for various durations, further leading to comparable P1 amplitudes.

The only factor affecting the P1 amplitudes observed in this study was subjective confidence. Stronger P1 amplitudes were associated with higher confidence for categorization, and the strongest P1 amplitudes were observed when participants were sure of the category of the stimulus. Interestingly, the N170 for faces was also affected by subjective confidence in a similar way, i.e. stronger N170 amplitudes being linked with higher confidence. These connections may indicate that the perception of low-level information (indexed by the stronger P1) is a prerequisite for the possible higher-level processing, e.g. categorization with high confidence (indexed by the stronger N170). Specifically, a stronger P1 may reflect that more attention was allocated on the stimulus and, therefore, the stimulus was more easily perceived with high subjective confidence.

In this study, we grouped N170 amplitudes by participants’ subjective confidence, which should reflect their subjective perception, and observed that the effects of durations on the N170 disappeared, suggesting that weaker N170 reflects the uncertainty of face perception but not the direct impact of limited stimulus durations. Since faces with shorter durations or more noise are both associated with lower stimulus visibility, it may be that similar differences in subjective perception, rather than the direct effect of noise, are linked with N170 differences in previous studies (Tarkiainen et al. 2002; Horovitz et al. 2004; Bestelmeyer et al. 2008; Németh et al. 2014). It is not a coincidence that subjective perception can dissociate trials generating full N170 from others, as the N170 is believed to be the index of subjective face perception/awareness (Rossion and Jacques 2011; Rossion 2014). These results provide further support to categorization of faces occurring in all-or-none fashion.

Given the relationships between subjective perception and the N170(/P1), we may need to reconsider the conclusions of other neural indicators drawn from previous studies where subjective perception might vary in different experimental conditions. For example, apart from the N170, stimulus visibility, which was controlled by shortening stimulus durations or adding noise, was also found to modulate the N1 (Haider et al. 1964; Bacon-Macé et al. 2005), N250 (Carbon et al. 2005; Jemel et al. 2010), P300/M300 (Carbon et al. 2005; Philiastides and Sajda 2006; Tanskanen et al. 2007), other ERP components (Jemel et al. 2010; Nasr 2010), and even neuroimaging responses (Bar et al. 2001; Natu et al. 2016). For these studies, caution is needed in drawing conclusions of a graded response before taking into account subjective perception of the stimuli.

We applied some relatively new statistical methods in this study. First, LMM incorporating both by-subject and by-stimulus random effects enabled us to generalize our results not only to similar participants but also to similar stimuli. Moreover, in addition to the conventional NHST, which can test whether there are significant differences but provides no mechanisms for endorsing the null hypothesis (Cohen 1994), we employed ET to test whether the effects of interest were compatible with the null hypothesis. ET is of great importance in providing evidence for accepting amplitude differences between durations being equivalent to null effects, which corroborates the all-or-none hypothesis.

In summary, our study showed that the “full” N170 is generated once a face is perceived with high subjective confidence, supporting the all-or-none hypothesis for the N170 generation. These results further suggest that categorization of faces also occurs in a similar all-or-none fashion.

## Supplementary Material

Supplementary_bhac101
