# Supplementary material for: All-or-none neural mechanisms underlying face categorization: evidence from the N170
Source: Cereb Cortex. 2022 Mar 15;33(3):777–93. doi: 10.1093/cercor/bhac101 (PMC9890453; doi:10.1093/cercor/bhac101)
Supplement: Supplementary_bhac101 [file supplementary_bhac101.zip › Supplementary_bhac101.pdf]

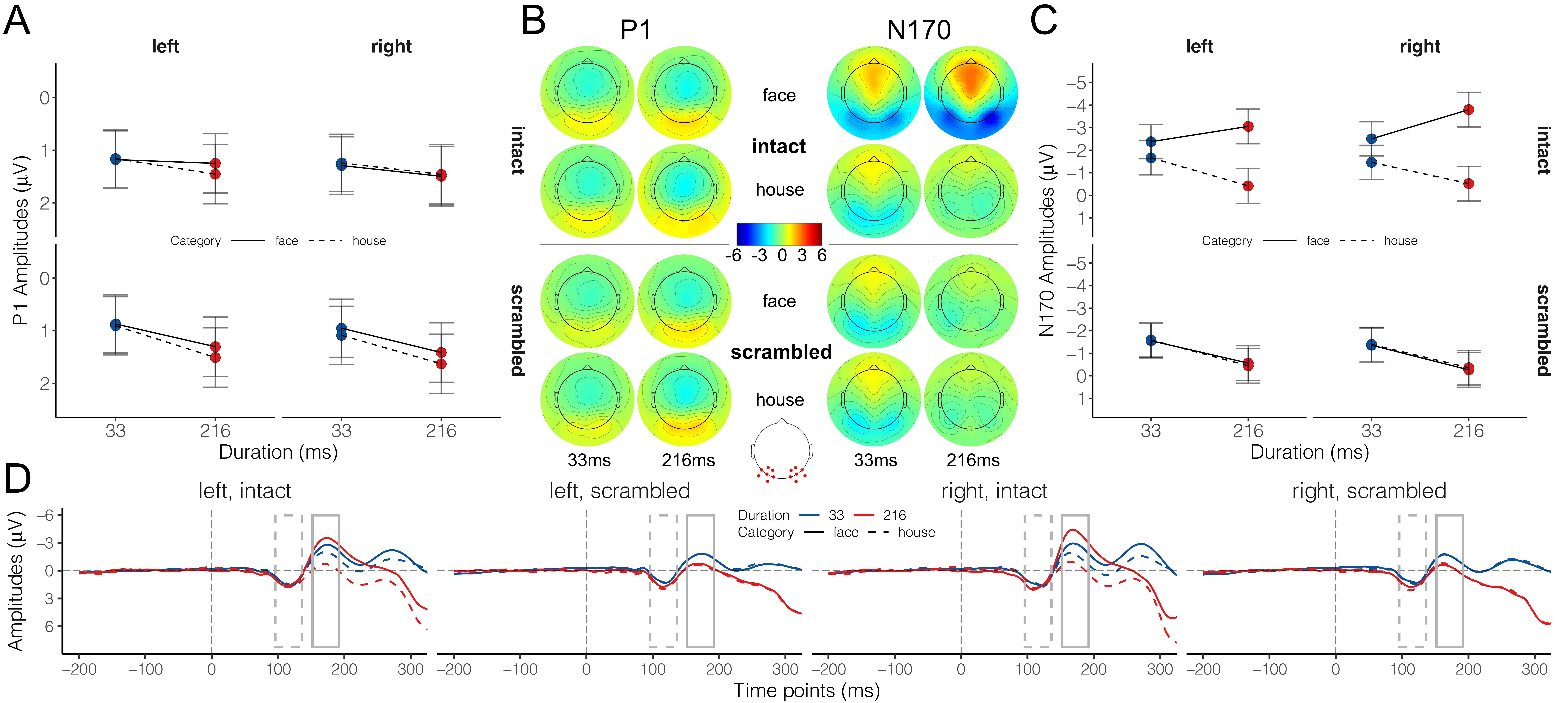

***Figure S1*. ERP results as a function of Hemisphere (left vs. right), Type (intact vs. scrambled), Category (faces vs. houses), and Duration (33ms vs. 216ms) across behaviour responses. A and C depict the estimated marginal means of P1 and N170 amplitudes, respectively; error bars represent 95% confidence intervals. B, Topographic maps corresponding to the P1 and N170 components. The red dots in the schematic brain denote the channel locations for both P1 and N170 on both hemispheres; the central electrode used for left hemispheres was E65 (PO7) and its neighbouring electrodes were E65, E70, E66, E59, E58, E64, and E69. The central electrode used for right hemispheres was E90 (PO8) and its neighbouring electrodes were E90, E96, E91, E84, E83, E89, and E95. D, The grand average ERP waveforms. The grey dashed rectangle denotes the integration windows for the P1 (96ms – 136ms), while the grey solid rectangle denotes the integration windows for the N170 (152-192ms).**

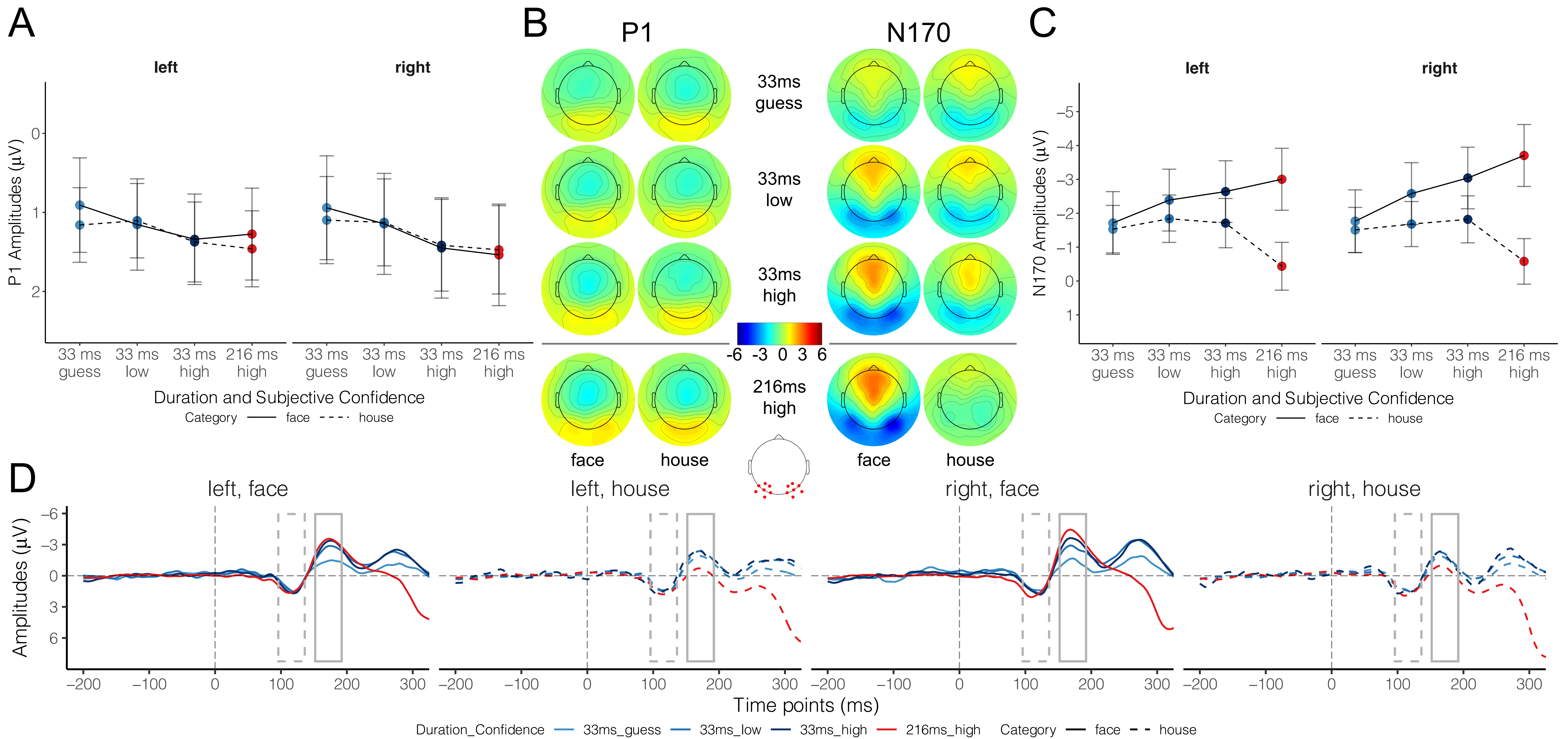

Figure S2. **ERP results for intact stimuli as a function of Hemisphere (left vs. right), Category (faces vs. houses), and Duration Confidence (33ms_guess, 33ms_low, 33ms_high, vs. 216ms_high). A and C depict the estimated marginal means of P1 and N170 amplitudes, respectively; error bars represent 95% confidence intervals. B, Topographic maps corresponding to the P1 and N170 components. The red dots in the schematic brain denote the channel locations for both P1 and N170 on both hemispheres; the central electrode used for left hemispheres was E65 (PO7) and its neighbouring electrodes were E65, E70, E66, E59, E58, E64, and E69. The central electrode used for right hemispheres was E90 (PO8) and its neighbouring electrodes were E90, E96, E91, E84, E83, E89, and E95. D, The grand average ERP waveforms. The grey dashed rectangle denotes the integration windows for the P1 (96ms – 136ms), while the grey solid rectangle denotes the integration windows for the N170 (152-192ms).**

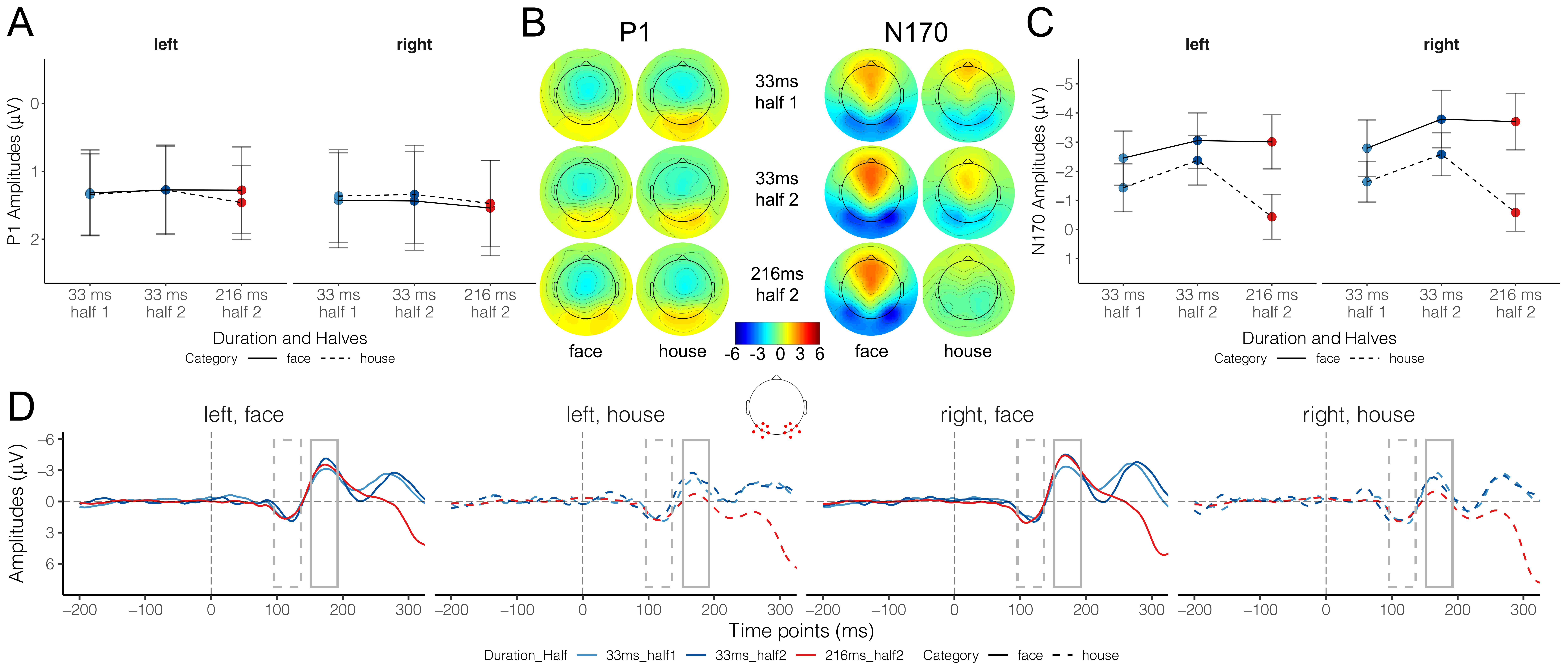

Figure S3. **ERP results for intact stimuli with high subjective confidence as a function of Hemisphere (left vs. right), Category (faces vs. houses), and Duration Half (33ms_half1, 33ms_half2, vs. 216ms_half2). A and C depict the estimated marginal means of P1 and N170 amplitudes, respectively; error bars represent 95% confidence intervals. B, Topographic maps corresponding to the P1 and N170 components. The red dots in the schematic brain denote the channel locations for both P1 and N170 on both hemispheres; the central electrode used for left hemispheres was E65 (PO7) and its neighbouring electrodes were E65, E70, E66, E59, E58, E64, and E69. The central electrode used for right hemispheres was E90 (PO8) and its neighbouring electrodes were E90, E96, E91, E84, E83, E89, and E95. D, The grand average ERP waveforms. The grey dashed rectangle denotes the integration windows for the P1 (96ms – 136ms), while the grey solid rectangle denotes the integration windows for the N170 (152-192ms).**

Table S1. **Statistical results of null hypothesis significance testing (NHST) and equivalence tests (ET) for the comparing P1 and N170 amplitudes, as well as the face-minus-house component, for different durations with subjective confidence (i.e., Duration Confidence). Each row displays the statistical results of one comparison pair for both left and right hemisphere electrodes. If the null hypothesis was rejected, only NHST results were reported; if the effect was practically equivalent to the null effects, only ET results were reported; and if the effects were inconclusive, both results were reported. The first row, for instance, denotes that the P1 differences between 17guess and 17low conditions were inconclusive for both hemisphere electrodes. (17guess: 17ms_guess; 17low: 17ms_low; 17high: 17ms_high; 200high: 200ms_high).**

**Component**	**Comparisons**	**Statistical results** (NHST and ET)	
		**left-hemisphere electrodes**	**right-hemisphere electrodes**
P1	17guess vs. 17low	**NHST**: *t*(37115.1) = -1.81, *p* = .42; **ET**: *t*(37115.1) = 1.88, *p* = .18	**NHST**: *t*(35346.6) = -1.51, *p* = .79; **ET**: *t*(35346.6) = 2.19, *p* = .09
	17guess vs. 17high	**NHST**: *t*(20687.5) = -3.14, *p* = .01, *b* = -0.43, 95% *CI* = [-0.80, -0.07]	**NHST**: *t*(15779.4) = -3.71, *p* = .001, *b* = -0.51, 95% *CI* = [-0.87, -0.15]
	17guess vs. 200high	**NHST**: *t*(36491.9) = -2.57, *p* = .06; **ET**: *t*(36491.9) = 0.94, *p* > .99	**NHST**: *t*(34614.7) = -4.19, *p* < .001, *b* = -0.60, 95% *CI* = [-0.97, -0.22]
	17low vs. 17high	**ET**: *t*(17691.8) = 2.86, *p* = .01, *b* = -0.19, 90% *CI* = [-0.45, 0.08]	**NHST**: *t*(13017.9) = -2.79, *p* = .03, *b* = -0.30, 95% *CI* = [-0.59, -0.02]
	17low vs. 200high	**ET**: *t*(37690.7) = 3.24, *p* = .004, *b* = -0.12, 90% *CI* = [-0.40, 0.16]	**NHST**: *t*(36493.1) = -3.35, *p* = .005, *b* = -0.39, 95% *CI* = [-0.70, -0.08]
	17high vs. 200high	**ET**: *t*(36231.4) = -3.96, *p* < .001, *b* = 0.07, 90% *CI* = [-0.20, 0.33]	**ET**: *t*(34348.2) = 3.76, *p* < .001, *b* = -0.09, 90% *CI* = [-0.35, 0.17]
face-minus-house P1	17guess vs. 17low	**NHST**: *t*(24749.2) = -1.79, *p* = .45; **ET**: *t*(24749.21) = 1.19, *p* = .70	**NHST**: *t*(25502.5) = -1.04, *p* > .99; **ET**: *t*(25502.5) = 1.93, *p* = .16
	17guess vs. 17high	**NHST**: *t*(5076.8) = -1.13, *p* > .99; **ET**: *t*(5076.8) = -1.47, *p* = .42	**NHST**: *t*(5541.5) = -0.99, *p* > .99; **ET**: *t*(5541.5) = 1.61, *p* = .32
	17guess vs. 200high	**ET**: *t*(31977.1) = 2.42, *p* = .05, *b* = -0.06, 90% *CI* = [-0.50, 0.37]	**NHST**: *t*(31001.3) = -1.23, *p* > .99; **ET**: *t*(31001.3) = 1.55, *p* = .36
	17low vs. 17high	**ET**: *t*(7492.4) = -2.43, *p* = .05, *b* = 0.08, 90% *CI* = [-0.33, 0.49]	**ET**: *t*(8082.1) = 2.82, *p* = .01, *b* = -0.02, 90% *CI* = [-0.43, 0.40]
	17low vs. 200high	**NHST**: *t*(38100.5) = 1.45, *p* = .88; **ET**: *t*(38100.5) = -1.64, *p* = .30	**ET**: *t*(37871.3) = 2.81, *p* = .01, *b* = -0.05, 90% *CI* = [-0.43, 0.34]
	17high vs. 200high	**NHST**: *t*(15301.1) = 0.84, *p* > .99; **ET**: *t*(15301.1) = -1.91, *p* = .17	**ET**: *t*(17940.9) = 2.59, *p* = .03, *b* = -0.03, 90% *CI* = [-0.47, 0.41]
N170	17guess vs. 17low	**NHST**: *t*(38713.2) = 4.84, *p* < .001, *b* = 0.68, 95% *CI* = [0.31, 1.04]	**NHST**: *t*(37587.1) = 5.85, *p* < .001, *b* = 0.82, 95% *CI* = [0.45, 1.18]
	17guess vs. 17high	**NHST**: *t*(29514.7) = 6.35, *p* < .001, *b* = 0.93, 95% *CI* = [0.54, 1.31]	**NHST**: *t*(22943.2) = 8.78, *p* < .001, *b* = 1.28, 95% *CI* = [0.89, 1.66]
	17guess vs. 200high	**NHST**: *t*(38302.6) = 8.78, *p* < .001, *b* = 1.29, 95% *CI* = [0.90, 1.68]	**NHST**: *t*(36887.3) = 13.23, *p* < .001, *b* = 1.94, 95% *CI* = [1.55, 2.33]
	17low vs. 17high	**NHST**: *t*(27206.8) = 2.15, *p* = .19; **ET**: *t*(27206.8) = -2.12, *p* = .10	**NHST**: *t*(20095.7) = 3.96, *p* < .001, *b* = 0.46, 95% *CI* = [0.15, 0.77]
	17low vs. 200high	**NHST**: *t*(38772.9) = 5.11, *p* < .001, *b* = 0.61, 95% *CI* = [0.30, 0.93]	**NHST**: *t*(37878.3) = 9.36, *p* < .001, *b* = 1.13, 95% *CI* = [0.81, 1.44]
	17high vs. 200high	**NHST**: *t*(38093.9) = 3.21, *p* = .008, *b* = 0.36, 95% *CI* = [0.06, 0.66]	**NHST**: *t*(36540.1) = 5.88, *p* < .001, *b* = 0.66, 95% *CI* = [0.37, 0.96]
face-minus-house N170	17guess vs. 17low	**NHST**: *t*(29293.0) = 2.09, *p* = .22; **ET**: *t*(29293.0) = -0.75, *p* > .99	**NHST**: *t*(20554.2) = 3.66, *p* = .002, *b* = 0.64, 95% *CI* = [0.18, 1.10]
	17guess vs. 17high	**NHST**: *t*(8673.0) = 3.52, *p* < .01, *b* = 0.75, 95% *CI* = [0.19, 1.31]	**NHST**: *t*(3906.4) = 4.60, *p* < .001, *b* = 0.96, 95% *CI* = [0.41, 1.52]
	17guess vs. 200high	**NHST**: *t*(34616.7) = 12.76, *p* < .001, *b* = 2.38, 95% *CI* = [1.89, 2.88]	**NHST**: *t*(29077.1) = 15.39, *p* < .001, *b* = 2.87, 95% *CI* = [2.38, 3.36]
	17low vs. 17high	**NHST**: *t*(11931.6) = 2.03, *p* = .25; **ET**: *t*(11931.6) = -0.64, *p* > .99	**NHST**: *t*(5675.1) = 1.74, *p* = .49; **ET**: *t*(5675.1) = -0.96, *p* > .99
	17low vs. 200high	**NHST**: *t*(38713.1) = 12.16, *p* < .001, *b* = 2.02, 95% *CI* = [1.58, 2.45]	**NHST**: *t*(37310.2) = 13.44, *p* < .001, *b* = 2.23, 95% *CI* = [1.79, 2.66]
	17high vs. 200high	**NHST**: *t*(19722.8) = 8.52, *p* < .001, *b* = 1.64, 95% *CI* = [1.13, 2.14]	**NHST**: *t*(11187.0) = 10.01, *p* < .001, *b* = 1.90, 95% *CI* = [1.40, 2.41]
